# Binding of Glutathione to Enterovirus Capsids Is Essential for Virion Morphogenesis

**DOI:** 10.1371/journal.ppat.1004039

**Published:** 2014-04-10

**Authors:** Hendrik Jan Thibaut, Lonneke van der Linden, Ping Jiang, Bert Thys, María-Dolores Canela, Leire Aguado, Bart Rombaut, Eckard Wimmer, Aniko Paul, María-Jesús Pérez-Pérez, Frank J. M. van Kuppeveld, Johan Neyts

**Affiliations:** 1 Department of Microbiology and Immunology, Rega Institute for Medical Research, University of Leuven, Leuven, Belgium; 2 Virology Division, Department of Infectious Diseases and Immunology, Faculty of Veterinary Medicine, Utrecht University, Utrecht, The Netherlands; 3 Department Medical Microbiology, Radboud University Nijmegen Medical Centre, Nijmegen Centre for Molecular Life Sciences, Nijmegen, The Netherlands; 4 Department of Molecular Genetics and Microbiology, School of Medicine, Stony Brook University, Stony Brook, New York, United States of America; 5 Department of Pharmaceutical Biotechnology & Molecular Biology, Vrije Universiteit Brussel, Brussel, Belgium; 6 Instituto de Química Médica (IQM-CSIC), Madrid, Spain; Purdue University, United States of America

## Abstract

Enteroviruses (family of the *Picornaviridae*) cover a large group of medically important human pathogens for which no antiviral treatment is approved. Although these viruses have been extensively studied, some aspects of the viral life cycle, in particular morphogenesis, are yet poorly understood. We report the discovery of TP219 as a novel inhibitor of the replication of several enteroviruses, including coxsackievirus and poliovirus. We show that TP219 binds directly glutathione (GSH), thereby rapidly depleting intracellular GSH levels and that this interferes with virus morphogenesis without affecting viral RNA replication. The inhibitory effect on assembly was shown not to depend on an altered reducing environment. Using TP219, we show that GSH is an essential stabilizing cofactor during the transition of protomeric particles into pentameric particles. Sequential passaging of coxsackievirus B3 in the presence of low GSH-levels selected for GSH-independent mutants that harbored a surface-exposed methionine in VP1 at the interface between two protomers. In line with this observation, enteroviruses that already contained this surface-exposed methionine, such as EV71, did not rely on GSH for virus morphogenesis. Biochemical and microscopical analysis provided strong evidence for a direct interaction between GSH and wildtype VP1 and a role for this interaction in localizing assembly intermediates to replication sites. Consistently, the interaction between GSH and mutant VP1 was abolished resulting in a relocalization of the assembly intermediates to replication sites independent from GSH. This study thus reveals GSH as a novel stabilizing host factor essential for the production of infectious enterovirus progeny and provides new insights into the poorly understood process of morphogenesis.

## Author Summary

Enteroviruses contain many significant human pathogens, including poliovirus, enterovirus 71, coxsackieviruses and rhinoviruses. Most enterovirus infections subside mild or asymptomatically, but may also result in severe morbidity and mortality. Here, we report on the mechanism of antiviral action of a small molecule, TP219, as an inhibitor of enterovirus morphogenesis. Morphogenesis represents an important stage at the end of the virus replication cycle and requires multiple steps, of which some are only poorly understood. Better understanding of this process holds much potential to facilitate the development of new therapies to combat enterovirus infections. We demonstrate that TP219 rapidly depletes intracellular glutathione (GSH) by covalently binding free GSH resulting in the inhibition of virus morphogenesis without affecting viral RNA replication. We discovered that GSH directly interacts with viral capsid precursors and mature virions and that this interaction is required for the formation of an assembly intermediate (pentameric particles) and consequently infectious progeny. Remarkably, enteroviruses that were capable of replicating in the absence of GSH contained a surface-exposed methionine at the protomeric interface. We propose that GSH is an essential and stabilizing host factor during morphogenesis and that this stabilization is a prerequisite for a functional encapsidation of progeny viral RNA.

## Introduction

Enteroviruses, belonging to the family of the Picornaviridae, are non-enveloped, icosahedral viruses with a positive, single-stranded genome. Enteroviruses comprise many important pathogens of humans and animals. Although most enterovirus infections subside asymptomatically or mildly, they can also result in severe morbidity and even mortality. Polioviruses cause paralytic poliomyelitis; rhinovirus infections are associated with exacerbations of asthma and chronic obstructive pulmonary disease and enterovirus 71 may cause life-threatening encephalitis, in particular in Asia. Also coxsackieviruses and echoviruses have been reported to cause acute clinical manifestations, including fulminant sepsis, aseptic meningitis and myocarditis [1]. Epidemiological studies strongly suggest a linkage between coxsackieviruses and the development of type 1 diabetes mellitus [2]. Apart from polio, no vaccines are available that can protect against enteroviral infections. No drugs have been approved so far for the treatment or prophylaxis of enterovirus infections [3].

The RNA genome of enteroviruses encodes four structural (VP1, VP2, VP3 and VP4) and seven nonstructural proteins (2A, 2B, 2C, 3A, 3B, 3C and 3D). Following receptor-mediated binding to the cell surface, the capsid is destabilized and the viral genome is delivered into the cytoplasm [4], [5]. Genomic RNA is then translated into a viral polyprotein which is processed into the structural capsid proteins and the non-structural proteins that are involved in the replication and production of new positive-strand RNAs via a negative-strand RNA intermediate. These newly synthesized positive-strand RNAs are produced on replication vesicles derived from the Golgi complex and trans-Golgi network (TGN) and either enter a new round of translation/replication or are packaged into capsid proteins to yield new infectious virus particles [6], [7].

Despite the fact that morphogenesis represents an important stage at the end of the virus replication cycle, many aspects of the molecular mechanisms dictating the assembly of viral particles remain obscure. It is known that this process occurs in a multi-tiered manner. In a first step, the capsid precursor P1, consisting of four structural components VP1 to VP4, is released from the P2–P3 polyprotein by a *cis*-cleavage, carried out by 2A^pro^ [8]. Studies with geldanamycin, a specific inhibitor of the cellular protein chaperone, heat shock protein 90 (Hsp90), have shown that by interacting with Hsp90, the nascent P1 is maintained in a processing-competent conformation and is protected from proteasomal degradation [9], [10]. This interaction with Hsp90 (and possibly Hsp70 and several cofactors), allows the precursor polyprotein P1 to be processed by 3CD^pro^ thereby yielding an immature structural unit, the protomer particle, containing one copy of VP0 (consisting of VP4 and VP2), VP3 and VP1 [11], [12]. Five of these protomers will then self-oligomerize into a pentameric particle. This self-assembly has been shown to be dependent on the modification of an N-terminal glycine residue of VP0 with myristate, stabilizing interactions at the fivefold axis between protomers forming pentameric subunits [13]–[16]. Further assembly of twelve pentameric subunits can generate a next higher-order particle, the empty capsid. It has been long a subject of debate whether newly synthesized progeny RNA is inserted into these preformed empty capsids or whether pentameric particles associate around an active replicating viral genome. An essential insight into this mechanism was provided by several studies suggesting a role for the non-structural protein 2C during encapsidation [17], [18]. Evidence was provided that viral encapsidation is coupled to genomic RNA synthesis resulting from a direct interaction between the RNA replication machinery and capsid proteins, possibly pentameric particles [19]. This hypothesis was extended by a recent study in which genetic and biochemical evidence was provided that newly synthesized positive-stranded genomes were encapsidated without an apparent involvement of an RNA packaging signal [20]. More specifically, a direct protein-protein interaction between 2C, as an essential component of the replication complex, and capsid protein VP3, possibly as part of a pentameric particle, was demonstrated. In this way pentameric particles will only condense at the site of replication around newly synthesized RNA genomes [19]–[21]. Further evidence for an interaction of protein 2C with capsid proteins during encapsidation was recently provided by a charged-to-alanine-scanning mutagenesis approach within protein 2C resulting in encapsidation-defective poliovirus and the identification of compensatory mutations in VP1 and VP3 [22]. In a last step and concomitantly with the encapsidation of genomic RNA, VP0 is processed into VP2 and VP4, probably by an RNA-dependent autocatalytic process, eventually yielding infectious virions [23].

Several studies provided evidence for a critical role of glutathione (GSH) during enterovirus morphogenesis [24], [25]. GSH is the most prevalent non-protein thiol in the animal cell and is involved in a multitude of cellular processes, including maintaining the cellular redox potential and detoxification of xenobiotics [where GSH serves as a cofactor in conjugation reactions catalyzed by glutathione peroxidases (GPx) and glutathione-S-transferases (GST)] [26], [27]. In addition, GSH has been shown to play a role in signal transduction, gene expression, cell proliferation and apoptosis by either maintaining a favorable redox status of the cell or by directly interacting with cysteine residues in polypeptidic chains [27]. An imbalance in GSH has been observed in various pathologies, including cancer, neurodegenerative disorders, cystic fibrosis and aging [28]. Through drug inhibition studies using buthionine sulfoximine (BSO), a known irreversible inhibitor of the γ-glutamylcysteine synthetase, (a key enzyme in the glutathione biosynthesis pathway), it was shown that GSH depletion results in a complete inhibition of the formation of infectious enterovirus progeny without affecting viral RNA and protein synthesis [24], [25]. The observed dependence on GSH during enterovirus morphogenesis was most likely not by affecting favorable cellular redox conditions since addition of other reducing agents could not substitute for GSH. The exact mechanism by which GSH is implicated in the formation of virus progeny remains obscure to date.

In this study, we describe the identification of TP219, a novel inhibitor of the assembly of several enteroviruses that acts by scavenging free GSH. By employing this inhibitor, we provide evidence that a direct interaction between VP1 and GSH is essential for the transition into or the stabilization of pentameric particles before assembling into higher-order particles. Furthermore, genetic analysis of coxsackievirus B3 (CVB3) escape mutants from GSH requirement demonstrated the relevance of a surface-exposed methionine at the interface of two protomers that may serve as a surrogate for GSH. Structural analysis of several naturally GSH-independent enteroviruses provided further evidence for the importance of this single residue in assembly. A role for GSH as a host factor during enterovirus morphogenesis is discussed.

## Results

### TP219 disrupt virus production without affecting viral RNA replication

**Figure 1:**
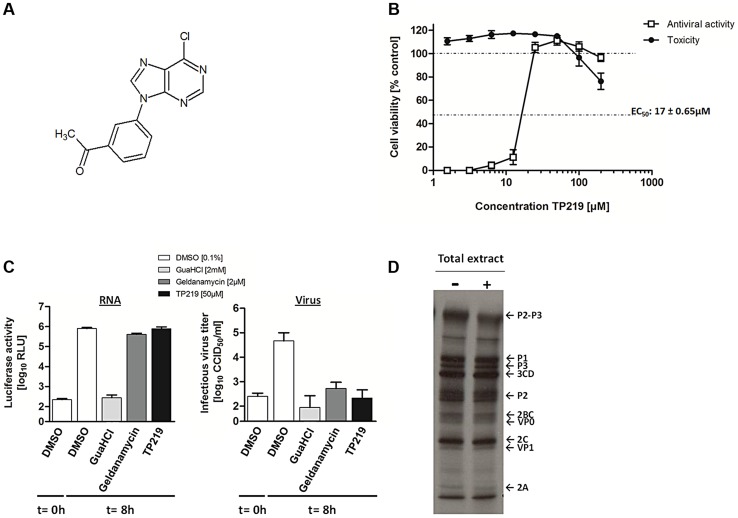
TP219 inhibits virus morphogenesis. (**A**) Structural formula of TP219. (**B**) Effect of TP219 on cell viability and CVB3-induced cytopathic effect in BGM cells. Toxicity (black circles) and CPE (white squares) was quantified by MTS assay at 3 d p.i. and expressed as percentage of untreated controls. Data are average values ± SD. (**C**) Analysis of the effect of TP219 on RNA replication and infectious virus titers. BGM cells were infected with RLuc-CVB3. The indicated compounds were added immediately after infection at the indicated concentrations. The enterovirus inhibitors GuaHCl and geldanamycin were included as controls. At 8 h p.i. intracellular viral RNA replication in the absence or presence of the indicated molecules was quantified by measuring luciferase activity (**C, left panel**). Lysates were used to determine infectious virus yields calculated by endpoint titration and expressed as the tissue culture 50% infectious dose per ml (TCID_50_) (**C, right panel**). Experiments were performed in triplicate and mean values ± SD are depicted. (**D**) Effect of TP219 on polyprotein processing. Cells were infected with CVB3 at MOI 50 and pulse-labeled with Methionine ^3S^[S] in the absence or presence of TP219. Subsequently, proteins were analyzed by SDS-PAGE.

We recently described a series of 9-arylpurines that inhibit *in vitro* enterovirus replication [29]. From this series, we selected TP219 (designated compound 26 in reference [29]) for further mechanistic studies (Fig. 1A). TP219 inhibits CVB3-induced CPE in BGM cells showing a 50% effective concentration of 17±0.65 µM with only little adverse effects on the host cell at high concentrations (Fig. 1B). TP219 was shown to exert antiviral activity against some enteroviruses (e.g. coxsackieviruses A16, A21 and A24, CVB3, echovirus 1 and 9 and poliovirus Sabin 3) but proved inactive against others (e.g. echovirus 11, poliovirus Sabin 1 and enterovirus 71) (Table 1). Interestingly, a cell type-dependent antiviral effect was observed. TP219 proved to be active against CVB3, echovirus 9 and CVA21 in Vero, BGM or MRC-5 cells, but not in HeLa or RD cells. EV71 and PV Sabin 1 remained insensitive when tested on Vero or BGM. Thus, TP219 inhibits the replication of a selection of enteroviruses in selected cell lines.

**Table 1:** Antiviral activity of TP219 against a selected panel of enteroviruses.

Species	Serotype (Strain)	Cell line	EC_50_ (µM)^a^
Enterovirus A	Coxsackievirus A16 (G-10)	MRC-5	2.7±0.76
	Enterovirus 71 (BrCr)	RD, HeLa, Vero	>100
Enterovirus B	Coxsackievirus A9 (Bozek)	HeLa	>100
	Coxsackievirus B3 (Nancy)	Vero	7.3±2.3
	Coxsackievirus B3 (Nancy)	BGM	17±0.65
	Coxsackievirus B3 (Nancy)	HeLa	>100
	Echovirus 1 (Farouk)	BGM	24±5.8
	Echovirus 9 (Hill)	MRC-5	3.3±1.2
	Echovirus 9 (Hill)	RD	>100
	Echovirus 11 (Gregory)	MRC-5	>100
Enterovirus C	Poliovirus 1 (Sabin)	HeLa, Vero, BGM	>100
	Poliovirus 3 (Sabin)	BGM	40±7.1
	Coxsackievirus A21 (Coe)	MRC-5	4.5±1.6
	Coxsackievirus A21 (Coe)	RD	>100
	Coxsackievirus A24 (Clinical)	MRC-5	2.7±0.87

Data are mean values ± SD from at least three independent experiments. The cytotoxicity values (CC_50_) were for all cell lines >100 µM.

a 50% effective concentration.

To explore the mechanism by which TP219 inhibits CVB3 replication, we systematically examined its effect on the different steps in the virus replication cycle. Towards that end, we used a reporter CVB3, expressing a *Renilla* Luciferase gene (RLuc-CVB3) placed between the 5′ UTR and the P1-coding region followed by a 3CD^pro^ cleavage site allowing for proteolytic processing of the polyprotein (Fig. 1C). BGM cells were infected with RLuc-CVB3 in the presence or absence of TP219. Geldanamycin and guanidine HCl (GuaHCl) were used as positive controls. GuaHCl is a known inhibitor of viral RNA replication; geldanamycin is a known inhibitor of Hsp90 and was previously reported to inhibit P1 maturation without affecting viral RNA replication [9]. CVB3 RNA replication was completely blocked in the presence of GuaHCl, but not in the presence of TP219 and geldanamycin (Fig. 1C left panel). Lysates of the infected cell cultures were subjected to end point titration to determine virus yields (Fig. 1C right panel). In the absence of compound, high virus titers were measured, indicating that the virus encapsidated the viral genome efficiently and was capable of infecting new cultures. In contrast, treatment with TP219 or geldanamycin resulted in a pronounced reduction of virus titers, indicating that, despite normal RNA replication levels, no infectious virus particles were formed. Thus, TP219 treatment did not affect early stages (such as attachment, entry or uncoating) or RNA replication. The fact that TP219 did not affect viral RNA replication indicates formation of intact and functional non-structural replication proteins. The defect in virus production might however be due to an adverse effect on 3C(D)^pro^-mediated proteolytic processing of structural capsid proteins. To test this possibility, CVB3-infected BGM cells were labeled with [^35^S]Met both in the absence or presence of TP219 between 5.5 and 6h p.i. (Fig. 1D) [9]. During this period CVB3 efficiently shuts off translation of cellular mRNA hence only viral proteins are radiolabeled. TP219 was shown not to directly affect 3C(D)^pro^-mediated proteolytic processing of the capsid-coding region as normal levels of VP0 and VP1 were observed (Fig. 1D). However, it cannot be ruled out that TP219 might have an indirect effect on proteolytic processing by affecting host cell metabolism which might require longer incubation periods.

Thus, these data demonstrate that TP219 does not inhibit entry, translation or RNA replication of CVB3, but interferes with virus morphogenesis.

### TP219 interferes with the transition of protomers into pentamers

**Figure 2:**
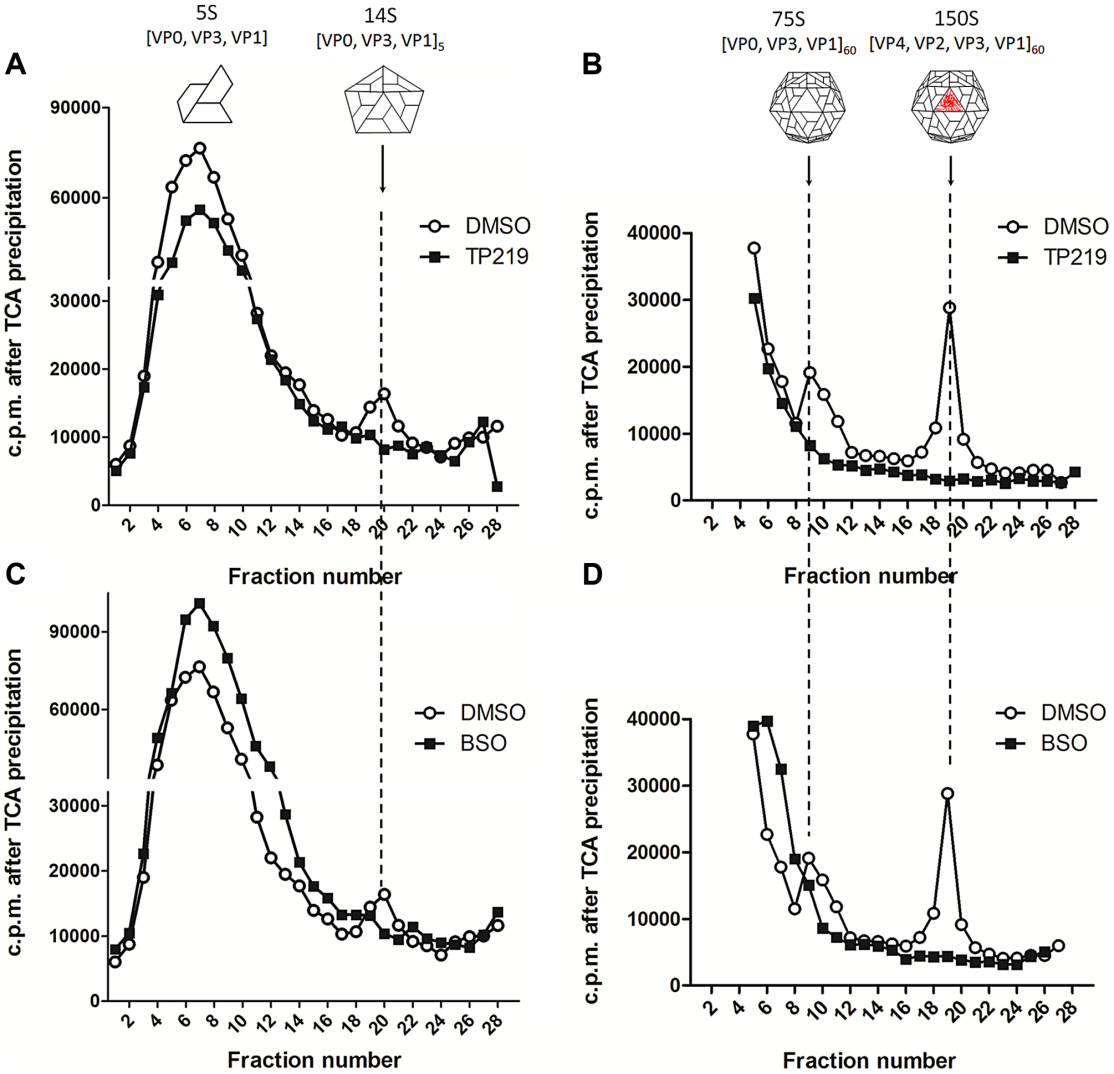
TP219- and BSO-treatment prevents the formation of 14S particles. Effect of TP219- (400 µM) (**A and B, black squares**), BSO- (2 mM) (**C and D, black squares**) and mock-treatment (**A–D, white circles**) on 5S, 14S, 75S and 150S assembly intermediates. Treated BGM cells were infected with CVB3 at a MOI of 10 and lysates were loaded onto 6 to 25% sucrose gradients for a separation of the 5S and 14S subunits (**A and C**) or onto 15 to 30% gradients for a separation of 75S and 150S subunits (**B and D**). Gradients were fractionated from the top, TCA-precipitated and counted by scintillation. Cartoon representations of the different assembly intermediates are indicated as well.

The effect of TP219 on the formation of different assembly intermediates (protomers [5S], pentamers [14S], empty capsids [75S] and mature virions [150S]) was next studied by sedimentation through a sucrose density gradient and analysis of gradient fractions by trichloroacetic acid (TCA) precipitation and liquid scintillation counting. In parallel, buthionine sulfoximine (BSO), a previously described enzymatic inhibitor of GSH synthesis and enterovirus morphogenesis, was included as a reference [25]. Mock (Fig. 2A–D), TP219- (Fig. 2A and 2B) or BSO- (Fig. 2C and 2D) treated BGM cells were infected with CVB3. 14S pentamers, 75S empty capsids and the 150S virions were readily detected in lysates of the CVB3-infected control cultures. Because of the high background near the top of the 6–25% sucrose gradient (Fig. 2A and 2C), 5S protomeric particles could not be distinguished. In the presence of TP219 or BSO, 14S, 75S or 150S assembly intermediates were not observed in the lysates of the infected cultures. Thus, TP219, akin to BSO, prevents the formation of 14S assembly intermediates and as a consequence the formation of consecutive higher-order particles.

**Figure 3:**
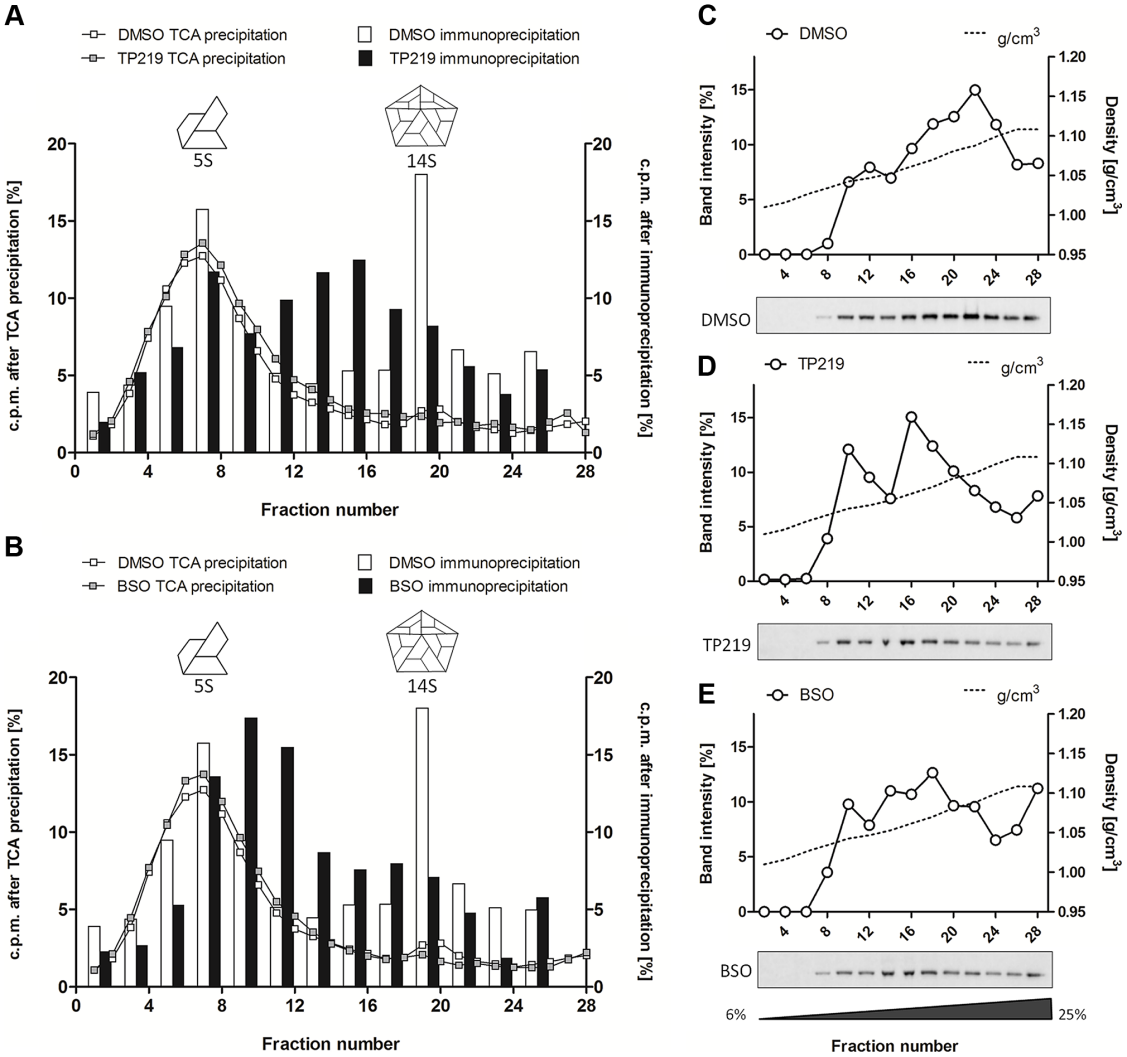
TP219- and BSO-treatment induces the formation of new assembly intermediates. Effect of TP219-, BSO- and mock treatment on the levels of 5S protomers and 14S pentamers as determined by immunoprecipitation (**A–B**) and immunoblotting analysis (**C–E**). (**A–B**) Two consecutive fractions of a 6 to 25% gradient were pooled, immunoprecipitated using polyclonal anti-enterovirus antibodies and assayed for radioactivity. Different immunoprecipitated fractions (bars) are overlaid on TCA-precipitated fractions (lines) (derived from Figure 2) and the amount of radioactivity is expressed as % of total signal to normalize for the incorporation efficiency. Immunoprecipitated fractions of untreated cultures (**A and B**) are indicated in white; TP219- (**A**) or BSO- (**B**) treated cultures in black. Cartoon representations of the 5S and 14S assembly intermediates are indicated as well. (**C–E**) Two consecutive fractions of a 6 to 25% gradient were pooled, TCA-precipitated and subjected to Western blotting analysis using a monoclonal anti-VP1 antibody. Both the immunoblot (below) and quantification of the immunoblot (top) of mock (**C**), TP219- (**D**) and BSO- (**E**) treated cultures are depicted. Sucrose density is indicated as well and expressed as g/cm^3^.

To allow a better dissection of the 5S and 14S viral particles and to reduce interference of labeled cellular and viral material, every two fractions of the 6–25% gradient were pooled and subjected to immunoprecipitation using polyclonal anti-CVB antibodies (Fig. 3A and 3B). Both 5S protomeric particles and 14S pentameric particles were detected in CVB3-infected control cultures. In analogy to the TCA-precipitated fractions, 14S peaks were largely reduced in the presence of either TP219 (Fig. 3A) or BSO (Fig. 3B). Also the levels of 5S protomeric particles were somewhat reduced in the presence of both compounds. Intriguingly, several peaks of unknown nature with a sedimentation coefficient varying from 5S to 14S appeared. To further corroborate the above, we next analyzed the effect of TP219 and BSO on the distribution of VP1 in 6–25% sucrose density gradient fractions (Fig. 3C–E). We therefore performed an immunoblotting analysis, using a monoclonal anti-VP1 antibody, on every two consecutive TCA-precipitated fractions and quantified the immunblot by densitometric analysis. Considering that protein composition (VP0, VP1 and VP3) remains intact during the transition of 5S into 14S particles, the data presented in Figure 3C–E clearly show that, akin to the immunoprecipitation experiments, TP219- (Fig. 3D) and BSO- (Fig. 3E) treatment affects the assembly pattern - as represented by VP1 - and results in a reduction of the levels of 14S pentameric particles as compared to the DMSO control (Fig. 3C).

Taken together, these data suggest that both TP219 and BSO affect the transition of protomeric subunits into pentameric subunits, or alternatively that they may affect the stabilization of pentameric particles, thereby resulting in the formation of several new assembly intermediates.

### TP219 rapidly depletes host cell glutathione levels via formation of an adduct

**Figure 4:**
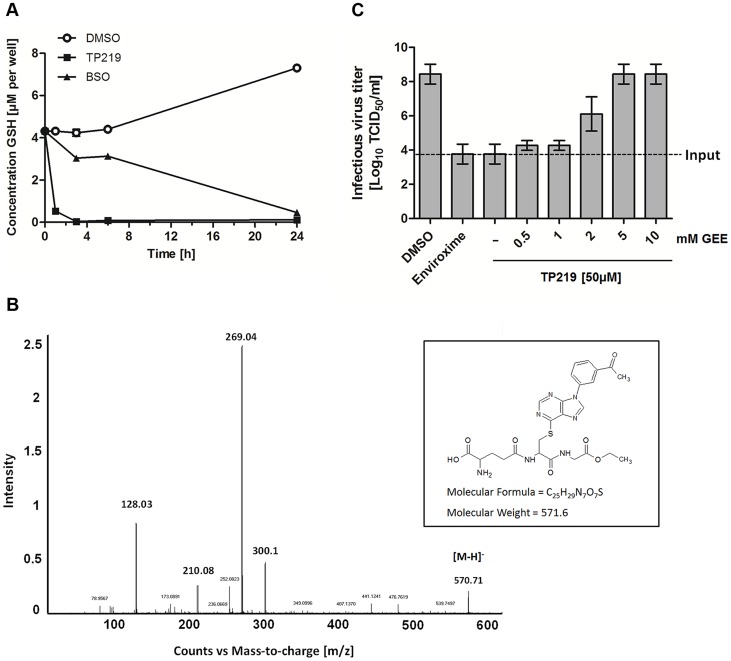
TP219 depletes intracellular GSH levels via a covalent bond. (**A**) Effect of TP219 (black squares) and BSO (white circles) versus control (black squares) on intracellular reduced (GSH) levels in BGM cells, quantified at various time points post incubation. (**B**) LC/MS/MS followed by collision induced dissociation analysis identified the ion [M-H]^−^ at m/z 570.71 and several fragment ions. Proposed structure of TP219-GEE adduct corresponding to [M-H]^−^ is provided as well. (**C**) The inhibition of infectious particle formation by TP219 can be reversed by a dose dependent exogenous addition of reduced GSH (GEE) to TP219-treated cell cultures. Control or TP219-treated (50 µM) cell cultures were co-incubated with varying concentrations of GEE, infected with CVB3 at an MOI of 5 and harvested 7 h pi. Virus titers were calculated by endpoint titration and expressed as the tissue culture 50% infectious dose per ml (Log_10_ TCID_50_). Data are average values ± SD.

We next explored whether, TP219 affects like BSO intracellular GSH levels. Intracellular levels of GSH in TP219- or BSO-treated BGM cells were monitored over a period of 24 hours. BSO treatment resulted in a progressive depletion of endogenous intracellular GSH levels and almost complete depletion (89±0.47%) was observed 24 hours post incubation (Fig. 4A). In contrast, already following one hour of incubation in the presence of TP219, the intracellular GSH content was reduced by more than 90%. GSH levels were completely depleted (99±0.048%) following 3 hours of incubation (Fig. 4A). Conversely, treatment of HeLa cell cultures with TP219 hardly resulted in any reduction in GSH levels (Fig. S1). In parallel, we also determined the total intracellular GSH concentration in BGM and HeLa cells, being 6.3±0.40 nmol/mg protein and 7.5±0.18 nmol/mg protein respectively. It is unlikely that this small difference accounts for the TP219 insensitivity of HeLa cells. However, it has been shown previously that tumor cell lines (such as HeLa cells) may have an increased GSH biosynthesis [30]. This could then result in a rapid replenishment of the depleted GSH pools following TP219-treatment. Within this context, it is likely that the differences in antiviral activity between HeLa (and most likely also RD) and BGM (and most likely also Vero and MRC-5) (see Table 1) cells are linked to GSH depletion or the lack thereof.

Depletion of GSH can be the consequence of an (i) increased GSH efflux (ii) increased cellular GSH oxidation or (iii) inhibition of GSH synthesis. TP219 did not markedly increase levels of extracellular GSH or intracellular GSSG (oxidized GSH) (data not shown). Moreover, the kinetic profile of GSH depletion in BSO treated cells differed from that in TP219-treated cells suggesting a mechanism of GSH depletion independent from inhibition of GSH synthesis. GSH is known to react as a nucleophile to form S-substituted conjugates even in the absence of catalyzing enzymes. The chlorine at position 6 of the chloropurine TP219 might be susceptible to a nucleophilic attack by the -SH group of GSH. The formation of such a conjugate can be determined by high performance liquid chromatography (HPLC) and mass spectrometry (MS) analysis. To explore this possibility, glutathione ethyl ester (GEE) (which was used as a surrogate because of its higher hydrophobicity and MS signal intensity compared to GSH) [31] was co-incubated with TP219. At different time points after incubation the samples were analyzed by HPLC. The peak corresponding to TP219 (retention time (rt) = 12.6 min) was significantly reduced in function of time with the concomitant formation of a new peak (rt = 10.7 min) (Fig. S2), which was analyzed by LC/MS/MS. Accurate mass determination (electrospray negative ion mode) indicated a peak with m/z 570.71 (corresponding to C_25_H_28_N_7_O_7_S) that was assigned as the conjugate [M-H]^−^ (Fig. 4B). Collision induced dissociation (CID) of this anion by MS/MS afforded fragment ions at m/z 300.11, 210.08 and 128.03 (Figure 4B), which have been proposed as an unambiguous identification of GEE trapped metabolites [32]. Taken together, these data suggest that TP219 forms an adduct with GSH, even in the absence of enzymes [31], [33].

To confirm the role of GSH in CVB3 morphogenesis, TP219-treated infected cells were supplied with exogenous GEE (which has better cell permeability than GSH). This resulted in a dose-dependent rescue of virus production (Fig. 4C). Since GSH is essential for maintaining a reduced cellular environment, GSH depletion might indirectly result in an antiviral status. Therefore, we studied the effect on virus infectivity of the reducing agents N-acetyl cysteine (NAC) and dithiothreitol (DTT) compensating for GSH depletion. In agreement with earlier observations made for BSO neither of them could serve as a surrogate for GSH (data not shown) [25].

### GSH-independent replication of CVB3 maps to a region in the capsid at the interface between two protomers

**Figure 5:**
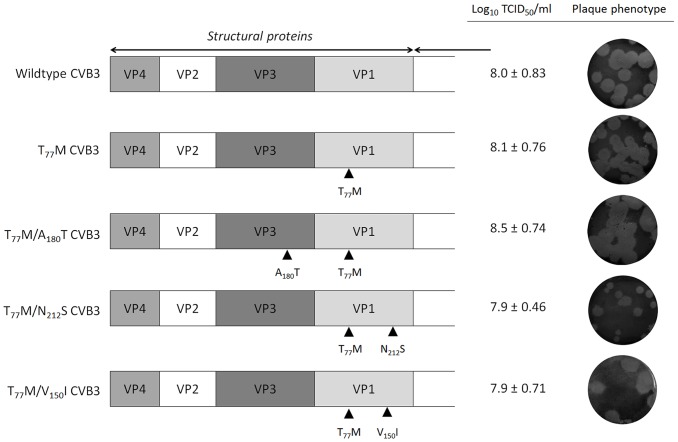
Growth phenotype of TP219-resistant CVB3 recombinants. The genomic organization of the structural proteins of CVB3 recombinants are illustrated on the left. Mutations in the genomic structure of P1 are shown by black rectangles. Virus infectious virus yields were calculated by endpoint titration and expressed as the tissue culture 50% infectious dose per ml (Log_10_ TCID_50_/ml). The plaque phenotype was determined using a plaque assay.

To identify whether we could drive CVB3 to replicate in the absence of GSH, three independent pools of CVB3 were repeatedly propagated in the presence of increasing concentrations of TP219, and thus decreasing concentrations of GSH. Following 17 sequential passages, several GSH-independent variants emerged that replicated (in contrast to the wildtype (wt) virus) effectively in the absence of GSH. To delineate the genetic basis for the observed resistance to GSH depletion, the complete nucleotide sequence of the three selected variants was determined and compared with the wt viral genome. In all three mutant pools a T_77_M mutation in VP1 in combination with one or two mutations in VP1 (V_150_I and N_212_S) or VP3 (A_180_T and K_135_R) (Table 2) was observed. A number of other mutations (in 2A and 3A) were not further considered, since they did not occur in all 3 variants. To assess the precise contribution of the identified mutations to the GSH-independent phenotype, recombinant viruses were engineered. Seven constructs were generated containing the identified mutations, either alone or combined, and RNA transcripts were transfected into BGM cultures. Mutation T_77_M alone (or combined), proved sufficient to bypass the need for GSH (Table 3). Constructs containing solely VP1 mutation V_150_I or N_212_S or VP3 mutation K_115_R still demonstrated a GSH-dependent phenotype. The T_77_M mutant CVB3 proved also cross-resistant with BSO (Figure S3). Virus titers and the plaque phenotype of the different resistant viruses were similar to that of the wt virus, which might be indicative for a comparable viral fitness, at least *in vitro* (Fig. 5). Only the T_77_M/N_212_S mutant produced smaller plaques than wt virus, which may suggest a role for the additional K_115_R mutation in VP3 that was not reintroduced into the genome of the T_77_M/N_212_S mutant.

**Table 2:** Amino acid mutations identified in three CVB3 pools following selection of GSH-insensitive variants.

		Nucleotide		
CVB3 protein	Amino Acid Substitution	Substitution	Position	Pool
VP3	K115R	AAG→AGG	1337	2
VP3	A180T	GCT→ACT	1531	1
VP1	T77M	ACG→ATG	1937	1,2,3
VP1	V150I	GTA→ATA	2155	3
VP1	N212S	AAC→AGC	2342	2
2A	E54K	GAG→AAG	2797	1,2
2A	E59V	GAA→GTA	2813	1,2
3A	I22V	ATT→GTT	4348	1,2
3A	K39R	AAA→AGA	4400	1,2

**Table 3:** Resistance pattern of recombinant CVB3 viruses carrying mutations in VP1 and VP3, either alone or combined.

		Mean EC_50_ [µM] ± SD of indicated antiviral molecule			
Clone	Mutation	TP219	Enviroxime	TBZE-029	BSO
	Wild-Type	7.3±2.3	0.84±0.23	4.4±0.21	87±7.8
1	T_77_M	>100	0.49±0.012	4.6±1.0	>2000
2	V_150_I	5.5±2.0	0.80±0.044	2.2±0.22	ND
3	N_212_S	6.5±0.17	0.38±0.18	3.1±0.67	ND
4	K_115_R	14±9.3	0.14±0.080	2.9±1.0	ND
5	A_180_T	ND	ND	ND	ND
6	T_77_M+V_150_I	>100	0.52±0.034	4.4±0.91	>2000
7	T_77_M+N_212_S	>100	0.21±0.051	2.8±0.74	>2000
8	T_77_M+A_180_T	>100	0.46±0.048	2.2±1.9	>2000

*Recombinant viruses were evaluated for inhibition by TP219, enviroxime, TBZE-029 and BSO*.

*Data represent EC_50_ values [µM] ± SD*.

*ND, not determined*.

### Structural analysis of GSH-independent enteroviruses

**Figure 6:**
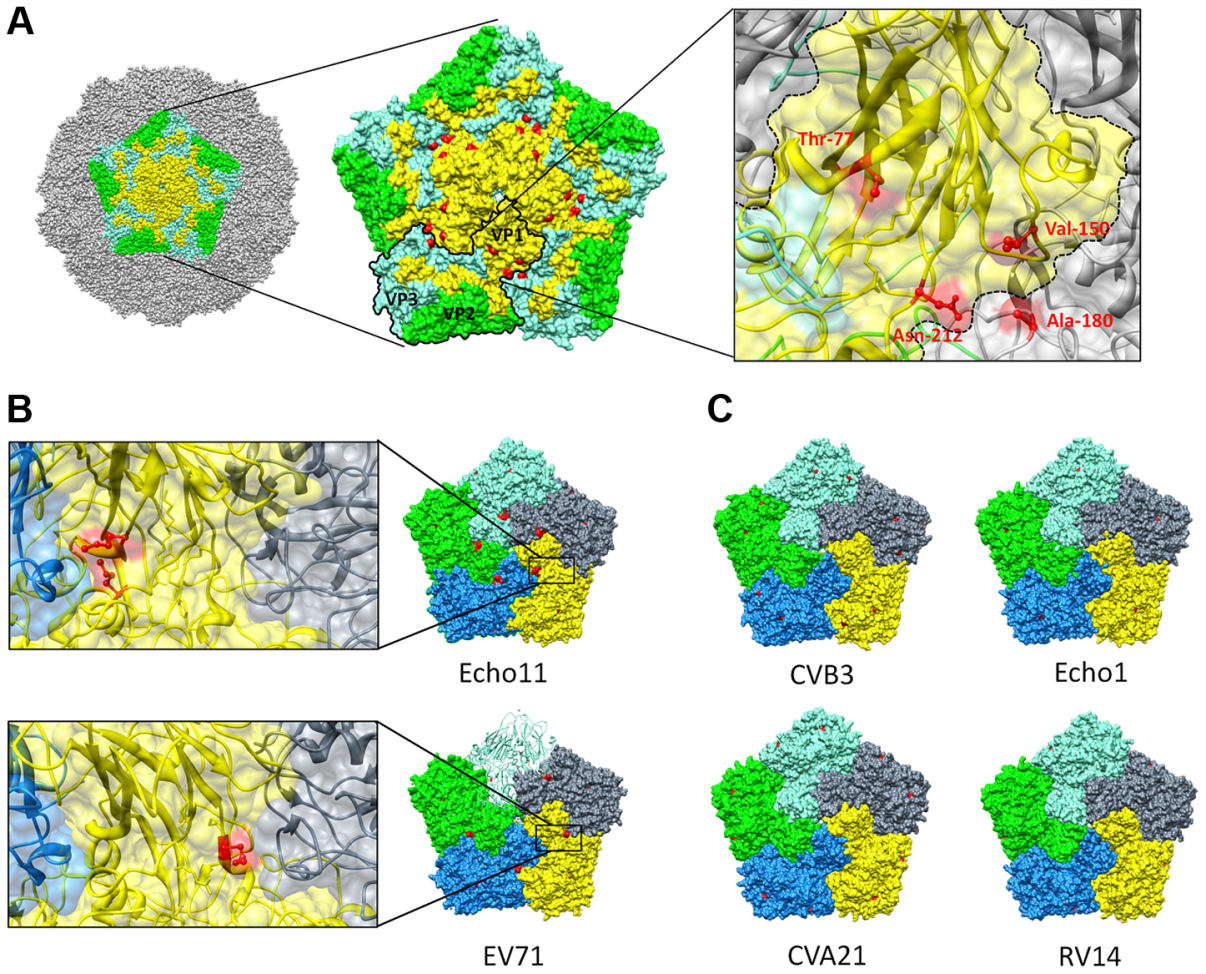
Resistance to GSH depletion maps to the interface between 2 protomers. (**A**) Surface rendered view of a single pentameric subunit (colored) in a whole capsid of CVB3 strain M (PDB 1COV). The remaining surface of the virion is colored grey. Viral proteins within a single pentameric unit are colored denoting VP1 (yellow), VP2 (green) and VP3 (aquamarine). A pentameric unit showing the location of the different mutations conferring GSH-insensitivity identified from the revertant screen (red) at the interface of 2 protomers. One protomer within the pentameric structure is outlined and an enlarged view on the antiparallel β-sheets in VP1 (colored yellow) and the location of the identified amino acids (colored red) on the interface between two protomers is provided as well. Comparison of pentamers of various GSH-insensitive (**B**) [echo11 (1h8t) and EV71 (4aed)] and GSH-sensitive (**C**) [CVB3 (1cov), echo 1 (1ev1), CVA21 (1z7s) and RV14 (1ro8)] enteroviruses. Surface representation of different protomers within a pentamer. Surface exposed methionines are indicated in red. An enlarged view on the location of the identified surface exposed methionines (colored red) on the interface between two protomers is also provided. Molecular graphics and analyses were performed with the UCSF Chimera package. Chimera is developed by the Resource for Biocomputing, Visualization, and Informatics at the University of California, San Francisco (supported by NIGMS P41-GM103311) [58].

The capsid of a mature virion consists of 60 protomers, each protomer consisting of a single copy of structural proteins VP1, VP2, VP3 and VP4, arranged on a pseudo *T* = 3 icosahedral surface [34]. Viral capsid proteins VP1, VP2 and VP3 are partially located at the outer surface of the capsid and adopt a similar eight-stranded antiparallel β-barrel fold which is conserved among enteroviruses. The antiparallel β-sheet barrel of VP1 harboring Thr-77 (the main mutation conferring GSH insensitivity that was identified in the revertant screen (Table 2), consists of βC, βH, βE and βF, and βB, βI, βD and βG which are packed face-to-face [35]. The Thr-77 is predicted to be a solvent exposed residue and is positioned in the βB, flanking the rim of the canyon closest to the fivefold axes [34]. Interestingly, the VP1 (Val-150 and Asn-212) and VP3 residues (Ala-180) are, akin to the Thr-77 residue, exposed on the surface of the CVB3 [M strain (1COV)] capsid and line the canyon surrounding the fivefold axis point (Fig. 6A). Although dispersed throughout the viral genome in the linear amino acid sequence, the identified amino acids all reside in close proximity to each other on the surface wall and more specifically opposing each other at the interface between protomeric subunits.

Next, we wondered whether the lack of activity of TP219 against several enteroviruses, including enterovirus 71 and echovirus 11 (Table 1), resulted from a natural resistance to GSH-depletion and whether this could be linked to a structural functionality. Within this context we analyzed the available crystallographic structures for the presence of a solvent exposed methionine, the main mutation conferring GSH-independence in CVB3 escape mutants. Rhinovirus 14 was also included in the analysis since this virus proved to be sensitive to GSH depletion in BSO-treated HeLa cells (data not shown). The naturally resistant viruses were found to be already carrying a solvent exposed methionine at the interface between two protomers (Fig. 6B). This methionine was not present in the GSH-dependent viruses (including echo1, CVA21 and RV14) (Fig. 6C). The assumed GSH-independence of echo11 and EV71 could be mapped to respectively a methionine at location 76 (positioned on the βB sheet) and 229 (positioned on the βH sheet) in VP1.

### Glutathione interacts directly with the capsid and is essential for morphogenesis/encapsidation

**Figure 7:**
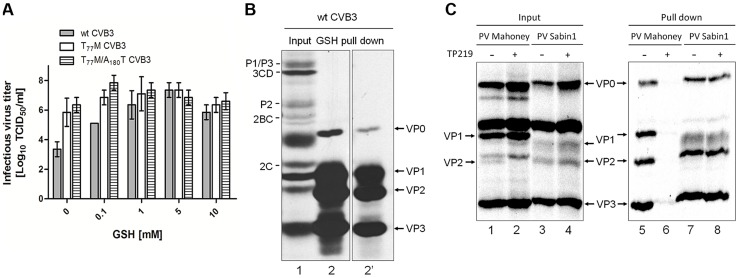
Direct interaction between VP1 and GSH. (**A**) Heat inactivation of 3×10^7^ TCID_50_/ml of wt, T_77_M and T_77_M/A_180_T CVB3 in the absence or presence of various concentrations of GSH. Following 30 minutes incubation at 46°C infectious virus titers were determined and expressed as Log_10_ TCID_50_/ml. (**B**) GSH pull down assay using glutathione-sepharose beads of CVB3-infected BGM cells. Labeling was done with Methionine ^3S^[S] and the lysate was analyzed by SDS-PAGE for the presence of viral proteins (input, lane 1). The lysate was loaded onto glutathione-sepharose beads and analyzed by SDS-PAGE (GSH pull down, lane 2). Lane 2′ shows a shorter exposure time of lane 2. (**C**) GSH pull down of PV Mahoney or PV Sabin1 infected Vero cells that were treated with TP219 or left untreated. Lysates were analyzed by SDS-PAGE (input, lanes 1–4) and loaded onto GSH Sepharose beads after which the pulled down material was analyzed by SDS-PAGE (pull down, lanes 5–8).

The assembly defect following GSH depletion likely results from the requirement for a direct interaction between GSH and the capsid. It has been described previously that capsid-binding molecules (e.g. pleconaril [36]) can protect against thermal-mediated inactivation by direct binding to and stabilizing of the virus particle [37]. We therefore incubated wt, T_77_M and T_77_M/A_180_T CVB3 at 46°C, a temperature detrimental for wt CVB3 infectivity, in the absence or presence of increasing concentrations of GSH. Wildtype CVB3 displays a temperature-sensitive phenotype, which is reversed upon addition of GSH (Fig. 7A). Interestingly, the infectivity of the T_77_M and T_77_M/A_180_T CVB3 remains almost unaffected at this temperature. These data strengthens the hypothesis that GSH interacts directly with the viral capsid thereby rescuing virus infectivity during heat inactivation. Thus, introducing a methionine at position 77 in VP1 generates a GSH-independent and temperature-insensitive phenotype.

To provide biochemical evidence for the interaction between GSH and the capsid, GSH-pull down assays were carried out (Fig. 7B). BGM cell cultures were infected with wt CVB3 after which the lysates were co-incubated with glutathione-sepharose beads and assayed for GSH affinity. Autoradiographic analysis clearly reveals that GSH is able to pull down VP0, VP1, VP2 and VP3 (lane 2 and 2′). Under these conditions of the gel, VP4 cannot be detected. Interestingly, the pull down mix contains both VP0 and VP2 which is indicative for the presence of both mature and precursor virus interacting with GSH.

To further corroborate this observation and to assess the GSH-binding properties of a GSH-independent enterovirus, Vero cells were infected with PV Mahoney, being a GSH-dependent virus (observed by Ma *et al.* in the accompanying paper) and PV Sabin 1, being a natural GSH-independent virus (Table 1). In addition, we assessed the effect of TP219-induced GSH depletion on this GSH-binding (Fig. 7C). As expected, no maturation cleavage of VP0 into VP2 and VP4 could be observed in TP219-treated cells infected with PV Mahoney (compare lanes 1 and 2), indicating that no infectious particles were formed, whereas this cleavage was readily observed for the GSH-independent PV Sabin 1 (compare lanes 3 and 4). Akin to CVB3, capsid proteins could be readily detected in the pull down of untreated cell lysates infected with both PV Mahoney (line 5) and PV1 Sabin 1 (line 7). However, upon TP219-induced GSH depletion capsid proteins of PV Mahoney, but not those of PV Sabin 1, lost their GSH-binding properties (compare lanes 6 and 8).

Together, these observations suggest that GSH interacts with enterovirus capsid proteins and that following GSH depletion, GSH-dependent viruses, unlike GSH-independent viruses, lose their GSH-binding properties.

### Glutathione is required for capsid proteins to localize in close vicinity of the replication sites

**Figure 8:**
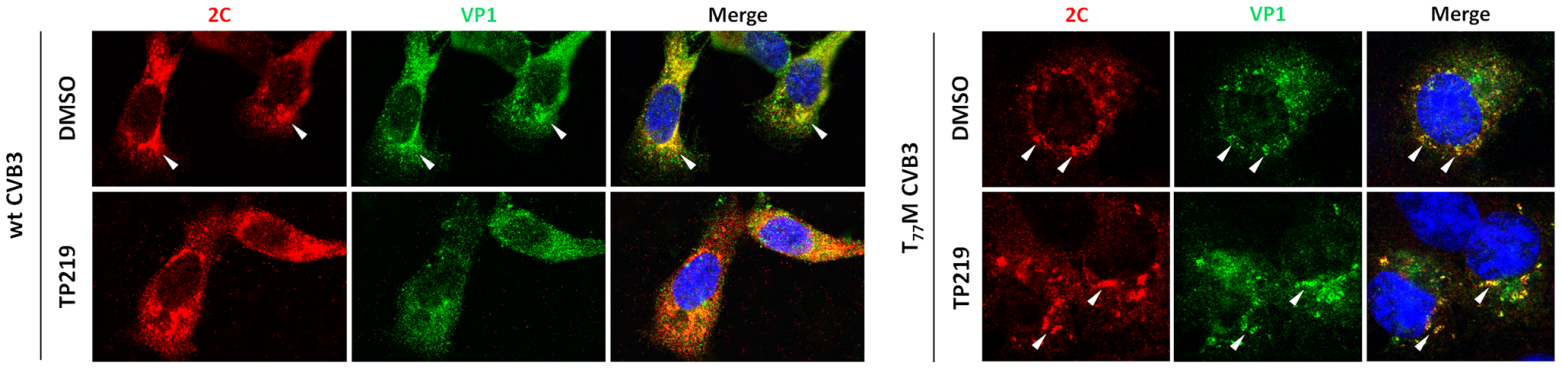
GSH depletion interferes with the interaction of VP1 and 2C. BGM cells were infected with wt or T_77_M CVB3 at a MOI of 10, in the absence or the presence of 50 µM TP219. Cells were fixed with saponin 0.5% at 5 h p.i. and costained with antibodies targeting 2C (and Alexa Fluor 568-conjugated secondary antibody (red color)) and VP1 (and Alexa Fluor 488-conjugated secondary antibody (green color)). Regions of colocalization are indicated by arrows.

It has been shown that (i) 5S and 14S particles associate with vesicular membranes containing the RNA replication complexes [38] and that (ii) encapsidation of progeny viral RNA is governed by a direct interaction between VP3 (possibly as part of these 5S or 14S particles) and replication protein 2C [20], [21]. More specifically a role for residue Glu-180 in VP3 (located at the interface of two protomers) was demonstrated to be an essential contact point with protein 2C. Intriguingly, the genetic evidence described above also implicates this region in GSH-independence. Therefore the impact of GSH depletion on the subcellular (co-)localization of VP1 and protein 2C in cells infected with either wt, T_77_M and T_77_M/A_180_T CVB3 was studied. Both in the absence and the presence of GSH, protein 2C of both wt and mutant CVB3 showed a distinct reticulated pattern in the perinuclear region (Fig. 8), suggesting that GSH depletion had no effect on the localization of protein 2C as part of the replication complex. This is also in line with the above observation that depletion of GSH levels does not inhibit viral RNA replication. In the absence of TP219 (i.e. in the presence of GSH), VP1 and 2C of both wt and the T_77_M CVB3 were observed to colocalize. Similar results were observed for T_77_M/A_180_T (Fig. S4). However, in the presence of TP219 (i.e. in the absence of GSH), VP1 of wt CVB3 showed a more dispersed pattern and no longer co-localized with protein 2C. Hence, GSH might be essential for localizing VP1, presumably as part of a 5S or 14S assembly intermediate, to the sites of replication allowing the encapsidation of newly synthesized viral RNA. Importantly, introducing a surface-exposed methionine in the capsid (T_77_M) resulted in a relocalization of VP1 to the replication sites even in the absence of GSH.

## Discussion

We here describe the discovery of TP219 as a novel inhibitor of the replication of several enteroviruses. We showed that TP219 forms a covalent adduct with GSH in an enzyme-independent way, thereby rapidly depleting intracellular GSH levels. As a consequence, the formation of infectious progeny virus is prevented without interfering with earlier events in the viral life cycle, such as entry, translation, proteolytic processing and RNA replication. By employing TP219, we further studied the details of virus assembly and present several lines of evidence that GSH is required during the transition of protomeric particles into pentameric particles. Inhibition of virus assembly following GSH depletion was not rescued by treatment with other reducing agents, indicating that a reducing environment is not a requirement for morphogenesis. We provided biochemical and microscopical evidence for a direct interaction between VP1 and GSH and that this interaction is essential for the translocation of assembly intermediates to the sites of replication. Furthermore, genetic and structural analysis of several GSH-independent viruses point towards the interface between protomers as a region critical for GSH binding/independence.

Capsid proteins VP1, VP2 and VP3 have the conserved β-sandwich motif consisting of eight-stranded antiparallel β-sheets packed face-to-face [35]. It has been previously suggested that modulation of the interface between protomers might be a mechanism shared by enteroviruses for the control of structural transitions during morphogenesis [39]. Within this context, it may be plausible that intact β-sheets at the protomeric interface are a prerequisite for morphogenesis and that (a) residue(s) within the β-barrel region interact with GSH, thereby modulating or stabilizing energetically favorable interactions that promote virus assembly. Indeed, fractionation studies revealed that in the absence of GSH the formation of pentameric particles was prevented without affecting the levels of protomers. These data suggest that GSH is needed for the transition into or the stabilization of pentameric particles required for assembly into higher-order particles.

Upon sequential passaging of (GSH-dependent) CVB3 in the presence of suboptimal levels of GSH, three GSH-independent variants were obtained. These virus isolates all contained a surface-exposed T_77_M mutation positioned in the βB-sheet of VP1 at the protomeric interface. Introducing this methionine into the wildtype CVB3 genome fully rescued the production of progeny virus, indicating that a single residue can mimic the presence of GSH. In addition, our antiviral data showed that some viruses, e.g. EV71 and echovirus 11, proved to be naturally insensitive to GSH-depletion. Genetic analysis of the available crystallographic structures of these GSH-insensitive viruses revealed the presence of a similar solvent-exposed methionine at the interface between two protomers. This further supports the hypothesis that GSH (or a surface-exposed methionine) may promote stabilization of β-sheets at the interface between protomers allowing further assembly into pentamers. Taking into account that GSH is an essential component of the cellular metabolism and is present at very high concentrations in mammalian cells, the natural presence of a surface exposed methionine in the capsid of several enteroviruses suggests that these viruses may already have evolved to assemble in the absence of glutathione despite the lack of a selective pressure (thus the absence of GSH). Whether the presence of a surface methionine represents a beneficial mutation conferring fitness advantage as also the question why most viruses do not already carry this residue remains to be answered.

Since addition of other sulfhydryl reducing agents such as DTT and NAC could not substitute for GSH, it seems unlikely that the apparent requirement for GSH during virus assembly results from an imbalance of the intracellular redox status or from an indirect effect on local pH which in turn has been reported to be important during assembly of bovine enterovirus *in vitro* [40]. This could indicate that GSH is directly rather than indirectly involved in the formation of pentameric particles. This was confirmed by employing a biochemical assay in which we demonstrated that GSH directly interacted with capsid proteins VP1, VP2, VP3 and VP0 of CVB3, PV (M) and PV Sabin 1. Further evidence for this direct interaction between the capsid and GSH was provided by heat inactivation experiments. The temperature sensitive (*ts*) phenotype of wt CVB3 is suppressed upon addition of GSH or changed to a temperature insensitive (non-ts) phenotype upon introducing a methionine at Thr-77. The fact that GSH (and methionine) is (are) capable of stabilizing virus infectivity following heat inactivation is in line with previous observations for capsid binding agents that, by direct binding to the virion, are also capable of rescuing infectivity following thermally induced conformational rearrangements [37], [41]. We therefore propose a critical role for GSH as a direct binding and stabilizing cofactor in the interaction between protomeric units assembling into pentameric particles. This hypothesis was further corroborated by biochemical assays showing a loss of GSH-binding properties of PV Mahoney capsid proteins following TP219 treatment and suggests that GSH depletion results in malformed protomeric particles that are unable to interact with GSH and also unable to fully assemble into pentameric particles. Remarkably, GSH-independent enteroviruses retain their GSH-affinity albeit this affinity is no longer required for assembly. Likewise, it has been shown that the temperature sensitivity of PV3/Sabin could be attributed to a S91F substitution in VP3 at the interface between protomers which resulted in the impaired formation of 14S pentameric particles at supraoptimal temperatures [39], [42]. Since non-ts revertants contained a number of second-site mutations, GSH or a surface-exposed methionine could in a similar way suppress conformational changes induced by heat inactivation.

It was recently shown that encapsidation of progeny viral RNA is directly linked to an interaction between VP3, (possibly as part of a pentameric particle), and non-structural protein 2C [20]. More specifically, Glu-180 in VP3 at the interface of 2 protomers was suggested to be an essential determinant for interaction with protein 2C. Further evidence for a direct interaction of 2C with capsid proteins was provided by a study in which several mutations in VP1 and VP3 were identified to compensate for a defect in encapsidation [22]. Despite the fact that 2C and capsid precursors (protomeric and pentameric particles) have been shown to co-localize at the surface of replication vesicles and can be cross-linked to viral RNA, it is still unknown whether 2C interacts directly with VP1 or VP3 or with a higher-order assembly intermediate [38], [43]. Our fractionation studies revealed almost exclusively protomers in TP219-treated cells. Strikingly, we observed an altered subcellular co-localization pattern of 2C and capsid protein VP1 (presumably as part of a protomeric particle) in TP219-treated cells by confocal analysis. On the contrary, the presence of a surface-exposed methionine resulted in a relocalization of VP1 to the sites of replication independent from GSH. Thus, GSH (or its “surrogate” T_77_M) proves essential for localizing assembly intermediates to the sites of replication. It is tempting to speculate that GSH is required for transition of protomers into pentamers and that pentamers are a prerequisite for a functional interaction with protein 2C. However, it cannot be formally ruled out that GSH depletion might also result in misfolded protomeric particles, resulting in a loss in the capacity to interact with 2C.

Intriguingly, GSH depletion following BSO-treatment in CVB3-infected HeLa cells was earlier shown not to affect the levels of protomeric and pentameric particles, but increased the levels of empty capsid particles [25]. In addition, Ma *et al.* observed an accumulation of pentameric particles and slightly faster sedimenting 150S particles in BSO-treated PV (M)-infected HeLa cell cultures [accompanying paper]. By contrast, our fractionation studies revealed the absence of pentameric and empty capsid particles in both BSO-treated and TP219-treated cells. It remains to be studied whether the use of strain CVB3/0 or PV1 (M) and HeLa cells instead of a Nancy CVB3 and BGM cells (our study) explains this difference.

Our data suggest that formation of infectious progeny involves the recruitment of assembly intermediates to a membranous surface containing the RNA replication complex. It has been demonstrated that the N-terminal glycine of VP4 is cotranslationally modified with a myristic acid linking each protomer within the pentameric structure to the two adjacent protomers at the 5-fold axes thereby stabilizing the structural elements within a pentameric unit [15]. Despite the fact that non-myristoylated protomeric particles can be found at the site of replication [13] and that purified non-myristoylated capsid proteins can assemble in solution into virus like particles [44], it has also been demonstrated that myristoylation might be a determinant for a correct membrane targeting of cytoplasmatically processed protomers [45]. However, since a N-myristoyl group confers only low hydrophobicity, additional (hydrophobic) factors may be required to improve membrane affinity [46], [47]. Within this perspective, we hypothesize that the tethering of myristoylated-protomers to membranous replication vesicles is enhanced by GSH or the presence of a methionine. Indeed, it has been recently suggested that glutathionylation of activated S100A9, a calcium-binding protein involved in fatty acid (FA) transport in neutrophils, might facilitate the interaction of S100A9 with the lipid environment during the translocation from the cytosol to the membrane, probably by increasing protein hydrophobicity [48]. Similarly, the presence of a surface exposed methionine, one of the most hydrophobic amino acids, has been described to be a key regulator for tight phospholipid membrane binding [49]–[51]. Albeit based on much speculation, methionine or GSH could then generate a hydrophobic surface region that increases membrane affinity following N-myristoyl-guided membrane targeting. Translocation of protomers to membranes may increase the change to encounter other protomers to form pentamers and, hence, the efficiency of assembling into higher order particles.

It remains unclear how GSH interacts with capsid proteins. Our data suggest that GSH is capable of interacting with capsid proteins both during and after maturation into infectious virus particles. This idea is supported by fractionation studies on sucrose gradients (showing that GSH is involved in the transition of 5S into 14S) and by heat inactivation (showing that GSH interacts with the infectious virion) and pull down experiments (demonstrating the presence of both VP0 and VP2 following GSH-pull down). In addition, in the accompanying paper Ma *et al.* demonstrate that capsid proteins can be readily detected following GSH-pull down of purified assembly intermediates. Despite the fact that these data suggest that GSH can interact with the maturing particle, GSH has not been observed in crystallographic structures for which several reasons could be hypothesized: the purification process might have resulted in the loss of GSH or GSH might be displaced by an unknown mechanism during/after morphogenesis. It might also be worth to explore in more detail whether unexplained electron densities exist in particles that may explain the presence of GSH. This might also be interpreted to mean that GSH interacts with capsid proteins probably in a non-covalent way which is corroborated by the fact that capsid proteins can be detected following pull down experiments using GSH-sepharose beads. In this system, the GSH ligand is attached to sepharose by a coupling via its sulfhydryl-group to the linker, implying that viral capsids show affinity for GSH without the apparent involvement of a SH-group. This also implies that (i) GSH does not form disulfide bonds with free cysteines in the viral capsid and (ii) opens the possibility that this free SH-group might be the determinant for a correct assembly process. Indeed, genetic analysis of GSH-independent viruses resulted in the identification of methionine – another sulfur containing amino acid - as the main determinant for GSH-independence. Further research is warranted to provide more details on this interaction and to clarify why GSH has not been observed thus far in the atomic structures of purified virus particles.

Reducing agents, including GSH and NAC have been frequently described to act as an non-conventional antivirals inhibiting the replication of several viruses, including HSV-1, HIV-1, influenza, and Sendai virus via various mechanisms [28]. This is the first paper, to our knowledge, that describes in detail the dependency of virus replication on GSH.

Glutathione is the most potent intracellular reductant in the cell and is implicated in various cellular processes, including signal transduction pathways, gene expression, cell proliferation or cell death [27]. Consequently, an imbalance in GSH has been associated with various pathologies, including neurodegenerative disorders and cystic fibrosis. In recent years a dysregulated GSH system has also been associated with the development of tumor chemoresistance, mostly by GST-mediated GSH conjugation to chemotherapeutic agents, resulting in less toxic GSH-drug complexes that are readily exported from the cell. It is expected that understanding these mechanisms will not only increase therapeutic response but will also decrease drug resistance [52], [53]. Within this context, TP219 could be a useful tool to study the direct impact of GSH depletion (by omitting long incubation periods as for BSO) on chemosensitivity in resistant tumors as well as the role for GSH in other processes such as virus replication.

## Materials and Methods

### Cells and reagents

BGM, HeLa, Vero, RD and MRC-5 cells were maintained in MEM (Gibco), supplemented with 10% fetal bovine serum (FBS) (Integro), 1% bicarbonate (Gibco) and 1% L-glutamine (Gibco). The synthesis of TP219 is described elsewhere [29]. Enviroxime was synthesized by Dr. G. Pürstinger (Institut für Pharmazie, Universität Innsbruck, Austria). Geldanamycin was from Biovision (Milpitas). L-Buthionine sulfoximine (BSO), guanidine hydrochloride (GuaHCl), N-acetyl cysteine (NAC), glutathione (GSH), glutathione ethyl ester (GEE) and dithiothreitol (DTT) were purchased from Sigma Aldrich.

### Viruses and antibodies

Coxsackievirus B3 (CVB3) was obtained by transfecting *in vitro*-transcribed RNA derived from plasmid p53CB3/T7 as previously described [54]. This plasmid contains a full length cDNA of CBV3 strain Nancy behind a T7 RNA polymerase promotor [55]. CVB3 expressing Renilla luciferase (RLuc-CVB3) was obtained by introducing the Renilla luciferase coding sequence between the 5′ untranslated region and the P1 coding region. The luciferase protein is followed by a 3CD cleavage site allowing posttranslational cleavage from the polyprotein by 3CD^pro^. Poliovirus Sabin 1 and 3 strains were from Prof. B. Rombaut (Vrije Universiteit Brussels, Belgium). Enterovirus 71 (BrCr), CVA16 (G-10) and CVA21 (Coe) were obtained from the National Institute for Public Health and Environment (RIVM, the Netherlands). CVA9 (Bozek), echovirus 9 (Hill) and echovirus 11 (Gregory) kindly provided by Dr. K. Andries at Janssen Pharmaceutica (Beerse, Belgium). For immunoprecipitation of sucrose gradients we used pooled rabbit polyclonal anti-enterovirus antibodies (including CVB1, 2 and 3) (Accurate Chemical and Scientific Corporation). For immunofluorescence or immunoblotting, we used as primary antibodies a mouse monoclonal anti-CVB3 VP1 (Dako) and a rabbit polyclonal anti-CVB3 2C (kindly provided by Prof. L. Whitton, Scripps Research Institute, USA). The secondary antibodies included Alexa Fluor 488-conjugated goat anti-mouse or 568-conjugated donkey anti-rabbit for immunofluorescence and goat-anti-mouse-IRDye680 for immunoblotting.

### Virus infections

For single cycle virus infections, virus was added to subconfluent cell layers and allowed to adsorb for 1 hour, after which virus was removed and fresh (drug-containing) medium was added to the cells. At 8 h p.i., cells were subjected to three cycles of freeze-thawing after which virus titers were determined by endpoint titration. Alternatively, cells were lysed to determine the intracellular Renilla luciferase activity with the Renilla Luciferase Assay System (Promega). For multicycle virus infections, cells grown to confluency in 96-well plates were subjected to serial dilutions of the compound and inoculated with the appropriate virus. After 3 days of incubation, cell viability was measured: after removal of the medium, 10% MTS/PMS (Promega) was added to each well and quantified spectrophotometrically at 498 nm in a microplate reader. The 50% effective concentration (EC_50_) was defined as the concentration of compound that inhibited virus-induced cytopathic effect formation by 50% and was calculated using logarithmic interpolation.

### Analysis of viral polyprotein processing *in vivo*

*In vivo* pulse-chase metabolic labeling studies were performed as described previously [56]. Briefly, BGM cells, grown to confluency in a 24-well plate were infected with CVB3 at a multiplicity of infection (MOI) of 50. At 5 h p.i., the cells were starved for methionine by replacing the medium with methionine-free medium for 30 min. Subsequently, the cultures were pulse-labeled in methionine-free medium containing ^35^S-labeled methionine for 30 min in the absence or presence of TP219 (50 µM final concentration). At 6 hours p.i., the cells were washed with PBS, lysed, and translation products were analyzed on a 12,5% polyacrylamide gel containing SDS, fixed, and then exposed to Kodak XAR film.

### Analysis of assembly intermediates by sucrose gradient ultracentrifugation

BGM cells grown to confluency in 75 cm^2^ flasks were infected with CVB3 at an MOI of 10. Following a one hour adsorption period, TP219 (400 µM) was added in complete medium. BSO-treated (2 mM) cells were pre-incubated for 48 hours. At 3.5 h post-infection, cells were starved for methionine by replacing the medium with methionine-free medium. Following a 30 minute incubation period, the cultures were pulse-labeled in methionine-free medium containing ^35^S-labeled methionine (20 µCi/ml). At 6 h. p.i., the cells were washed three times with 1% of ice-cold unlabeled methionine. Following three cycles of freeze-thawing, equal volumes of supernatants were loaded onto 6 to 25% sucrose gradients (wt/vol) for a separation of the 5S and 14S subunits or onto 15 to 30% (wt/vol) for a separation of 75S and 150S subunits. Gradients were centrifuged at 39,000 rpm in a Beckman SW40 rotor for 16 h (6 to 25% gradients) or 2 h 15 min (15 to 30% gradients). Gradients were fractionated in 400 µl aliquots from the top and precipitated using trichloroacetic acid (TCA). The radioactivity present in each fraction was quantified by liquid scintillation counting.

### Immunoprecipitation of viral particles

Immunoprecipitation studies were performed as previously described [57]. Briefly, every two consecutive samples of a sucrose gradient were pooled and immunoprecipitated using rabbit polyclonal anti-CVB3 antibodies. Following a 1 hour incubation period at 4°C, a 10% suspension of fixed Staphylococcus aureus strain Cowan I was added. Following 30 min incubation at 4°C, the samples were centrifuged for 5 min at 4°C. The immunoprecipitates were resuspended in NET buffer (150 mM NaCl, 5 mM EDTA, 50 mM Tris, 0.05% Triton X-100, 0.1% bovine serum albumin, 0.2% methionin and 0.1% cysteine) and centrifuged for 5 min at 4°C. The immunoprecipitates were resuspended in 2% SDS and boiled for 5 min. Finally, following centrifugation, the resulting supernatants were assayed for radioactivity by liquid scintillation counting.

### Immunoblotting analysis of assembly intermediates

TCA-precipitated fractions of a sucrose gradient were subjected to SDS-PAGE, transferred to a PVDF membrane, incubated with primary antibody and secondary antibodies and scanned using an Odyssey Imager (Li-COR). Densitometry was performed on immunoblot images using the ImageJ gel analysis tool.

### Detection of intracellular reduced and oxidized glutathione

Cells grown to confluency in 96-well plates were incubated in the presence or the absence of 50 µM TP219. At the indicated times post treatment with reduced glutathione (GSH) and oxidized glutathione (GSSG) levels were measured using the GSH/GSSG-Glo Assay (Promega) according to the recommendations of the manufacturer.

### High-performance liquid chromatography (HPLC) and mass spectrometry (MS) analysis

TP219 (10 µM) and glutathione ethyl ester (2 mM; pH 7.4) were co-incubated at 37°C. Samples were taken at four different time points (0, 3, 6 and 24 h) and analyzed by HPLC. HPLC spectra were recorded on a Agilent 1120 compact LC instrument using a diode array detector (230 to 400 nm) and an analytical ACE 5 C18-300 column (4.6 mm×15 cm) at a flow rate of 1 mL/min. Solvents used were acetonitrile (solvent A), and H_2_O (0.05% TFA) (solvent B). The gradient was used as follows: a 2 to 10% in 7 min (solvent A) followed by a 10 to 100% in 8 min (solvent A). Mass spectrometry analysis was performed using a HPLC-Waters 12695 connected to a Waters Micromass ZQ spectrometer (column: Sunfire C18, 4.6×50 mm, 3.5 µm particle size). Solvents used were acetonitrile (solvent A) and H_2_O (0.1% formic acid) (solvent B). A 15 to 95% gradient (solvent A) was carried out in 5 min (1 mL/min). Electrospray ionization, positive ion mode; capillary voltage 3.5 kV, cone voltage 30 V.

### Isolation of resistant CVB3

TP219 resistant (GSH-independent) CVB3 was generated by culturing CVB3 in the presence of increasing concentrations of TP219. After 3 days of incubation, lysates from those cultures that exhibited CPE in the presence of the highest concentration of compound were collected and were used to infect new cell monolayers for successive rounds until viral replication was observed at concentrations that do not allow replication of wt virus. Subsequently, viral RNA was isolated (Macherey-Nagel) and both DNA strands were sequenced [cycle-sequencing method (ABI Prism Big Dye Terminator Cycle Sequencing Ready Reaction Kit)] using an ABI 373 Automated Sequence Analyzer (Applied Biosystems)

### Site-directed mutagenesis

Mutant CVB3 clones were constructed, containing either single or multiple amino acid replacements at diverse positions in the capsid region. The eight clones were designated CVB3[T_77_M], CVB3[V_150_I], CVB3[N_212_S], CVB3[K_115_R], CVB3[A_180_T], CVB3[T_77_M/V_150_I], CVB3[T_77_M/N_212_S], CVB3[T_77_M/A_180_T]. The following synthetic oligonucleotides (and their complementary reverse oligonucleotides) were used for site-directed mutagenesis of the single mutants: (CVB3[T_77_M]) 5′- A TGT AGG TCA GCA TGC GTG TAC TTT ATG GAG TAT AAA AAC TC -3′, (CVB3[V_150_I]) 5′- GTA CCA CCA GGT GGA CCT ATA CCA GAT AAA GTT GAT T -3′, (CVB3[N_212_S]) 5′- GC ATC AAC ACG CTA AAC AGC ATG GGC ACG CTA TAT G -3′, (CVB3[K_115_R]) 5′- CA CAT TGG TCA GGC AGC ATA AGG CTT ACG TTT ATG TTC T -3′; (CVB3[A_180_T]) 5′- ACA CAC TAC CGG TTT GTT ACT TCA GAT GAG TAT ACC G -3′; The mutated sequences are underlined. Site-directed mutagenesis was performed with plasmid p53CB3/T7 using the XL Blue Large Site-directed mutagenesis kit (Stratagene), according to the manufacturer's instructions. After mutagenesis, the individual clones were verified by sequencing. The double mutants CVB3[T_77_M/V_150_I] and CVB3[T_77_M/A_180_T] were generated by cloning the single mutant CVB3[V_150_I] and CVB3[A_180_T] into CVB3[T_77_M] using respectively enzymes EcoRI/SpeI and BglII/SpeI. The double mutant CVB3[T_77_M/N_212_S] was generated by introducing the mutation at position N_212_S into CVB3[T_77_M] using mutagenesis. Next, enzymes BglII/SpeI were used to isolate the fragment containing the desired mutations, and reintroduce in an original, non-mutagenized clone of the same plasmid p53CB3/T7. From these mutants, RNA transcripts and infectious viruses were generated as described previously [54].

### Viral plaque assays

For determination of viral plaques, BGM cells, grown to confluency in six-well plates, were infected with the appropriate virus at 37°C. After 1 h, the virus was removed and the growth medium was replaced with agarose. Giemsa solution was used to stain the cells.

### Heat inactivation experiments

Wildtype, T_77_M and T_77_M/A_180_T CVB3 were incubated with GSH for 15 min at room temperature and subjected to heat treatment by incubation for 30 min at 46°C either in the absence or presence of various concentrations of GSH after which virus titers were determined by endpoint titration.

### GSH-pull down experiments

TP219-treated BGM or Vero cells were infected with CVB3, PV Mahoney or PV Sabin 1 (MOI 10), labeled with ^35^S-Translabel 4–6 hr post-infection and harvested in TNM buffer (10 mM Tris pH 7.5, 10 mM NaCl, 1.5 mM MgCl_2_, 0.1% Tween). After freeze-thawing, the viral proteins were analyzed by SDS-PAGE or incubated with Glutathione sepharose 4B beads (GE Healthcare life science) overnight at 4°C. The GSH-beads were washed five times with TNM buffer and boiled in 1× SDS-PAGE sample buffer and analyzed by SDS-PAGE.

### Confocal microscopy

Cells were grown to subconfluency in a 8-well chamber slide (Lab-tek, II, Nunc, Germany) and infected with the appropriate virus at an MOI of 10. After 1 h, virus was removed and cells were treated with TP219 (50 µM). After 5 h, cells were fixed with 4% paraformaldehyde and permeabilized with PBS containing 0.5% Saponin. Subsequently, cells were stained with primary and fluorescently-labeled secondary antibodies. Nuclei were stained with DAPI. Cells were visualized using a confocal laser scanning microscope (LCSM, Leica Microsystems, Germany).

## Supporting Information

Figure S1 **TP219 has no effect on intracellular GSH levels in HeLa cells.** Effect of TP219 (white bars) and BSO (grey bars) on intracellular reduced (GSH) levels in BGM cells, expressed as percentage untreated control at various time points post incubation.
(TIF)

Figure S2 **HPLC chromatogram of a new conjugate.** TP219 and GEE were co-incubated for 6 h and analyzed by HPLC. Samples were analyzed at two wavelengths λ = 254 nm (**A**) and λ = 230 nm (**B**).
(TIF)

Figure S3 **Cross-resistance to BSO.** BSO inhibits formation of infectious virus particles of wild-type (white bars) but not of T_77_M (grey bars) CVB3 in a dose-dependent manner. Virus titers were calculated by endpoint titration and expressed as tissue culture 50% infectious dose per ml (Log_10_ TCID_50_/ml). Data are average values ± SD.
(TIF)

Figure S4 **GSH depletion interferes with the interaction of VP1 and 2C.** BGM cells were infected with T_77_M/A_180_T CVB3 at a MOI of 10, in the absence or the presence of 50 µM TP219. Cells were fixed with saponin 0.5% at 5 h p.i. and costained with antibodies targeting 2C (and Alexa Fluor 568-conjugated secondary antibody (red color)) and VP1 (and Alexa Fluor 488-conjugated secondary antibody (green color)). Regions of colocalization are indicated by arrows.
(TIF)
