# Supplementary figures and images for: Binding of Glutathione to Enterovirus Capsids Is Essential for Virion Morphogenesis
Source: PLoS Pathog. 2014 Apr 10;10(4):e1004039. doi: 10.1371/journal.ppat.1004039 (PMC3983060; doi:10.1371/journal.ppat.1004039)

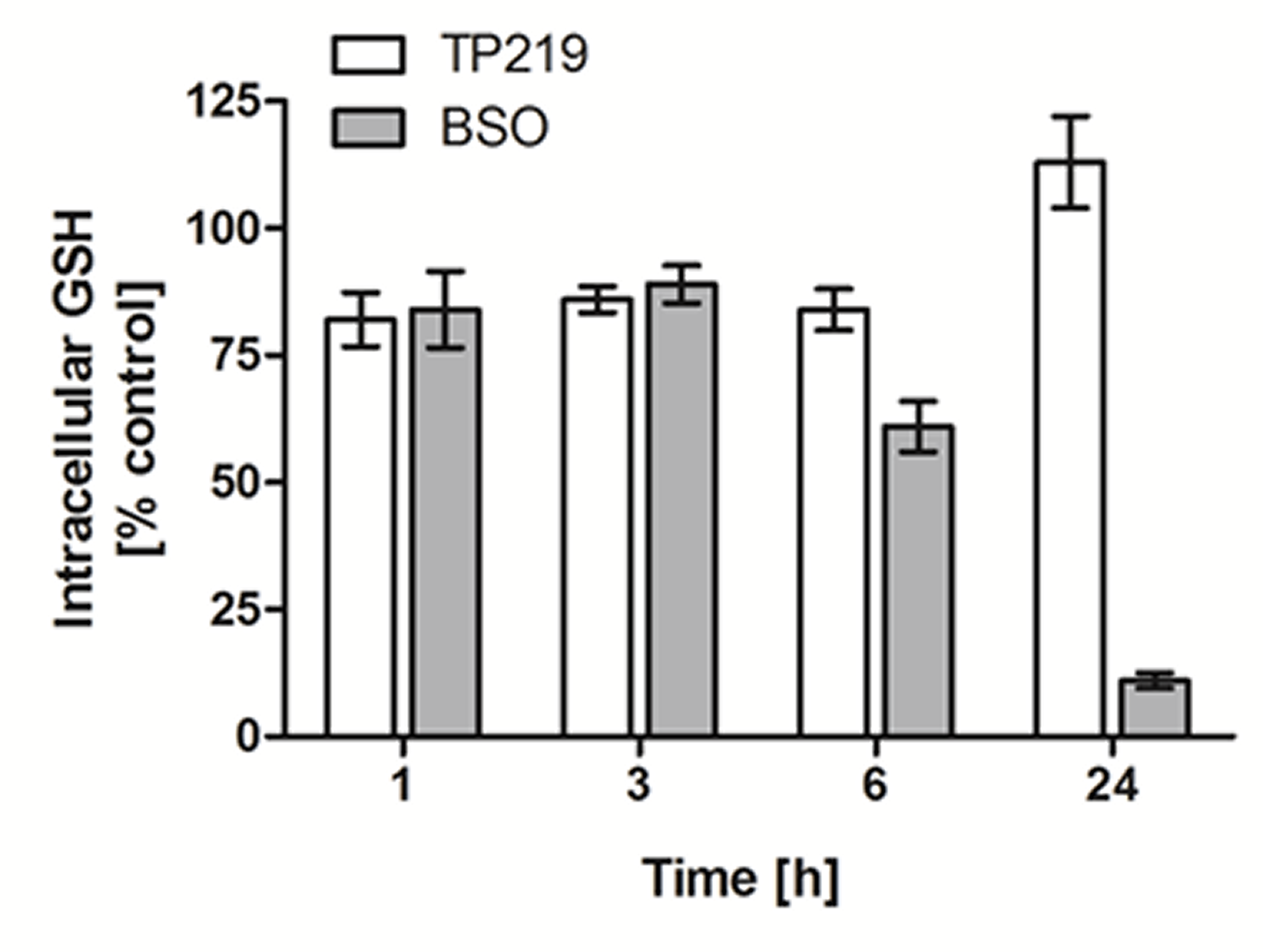

Supplement: Figure S1 — TP219 has no effect on intracellular GSH levels in HeLa cells. Effect of TP219 (white bars) and BSO (grey bars) on intracellular reduced (GSH) levels in BGM cells, expressed as percentage untreated control at various time points post incubation. (TIF) [file ppat.1004039.s001.tif]

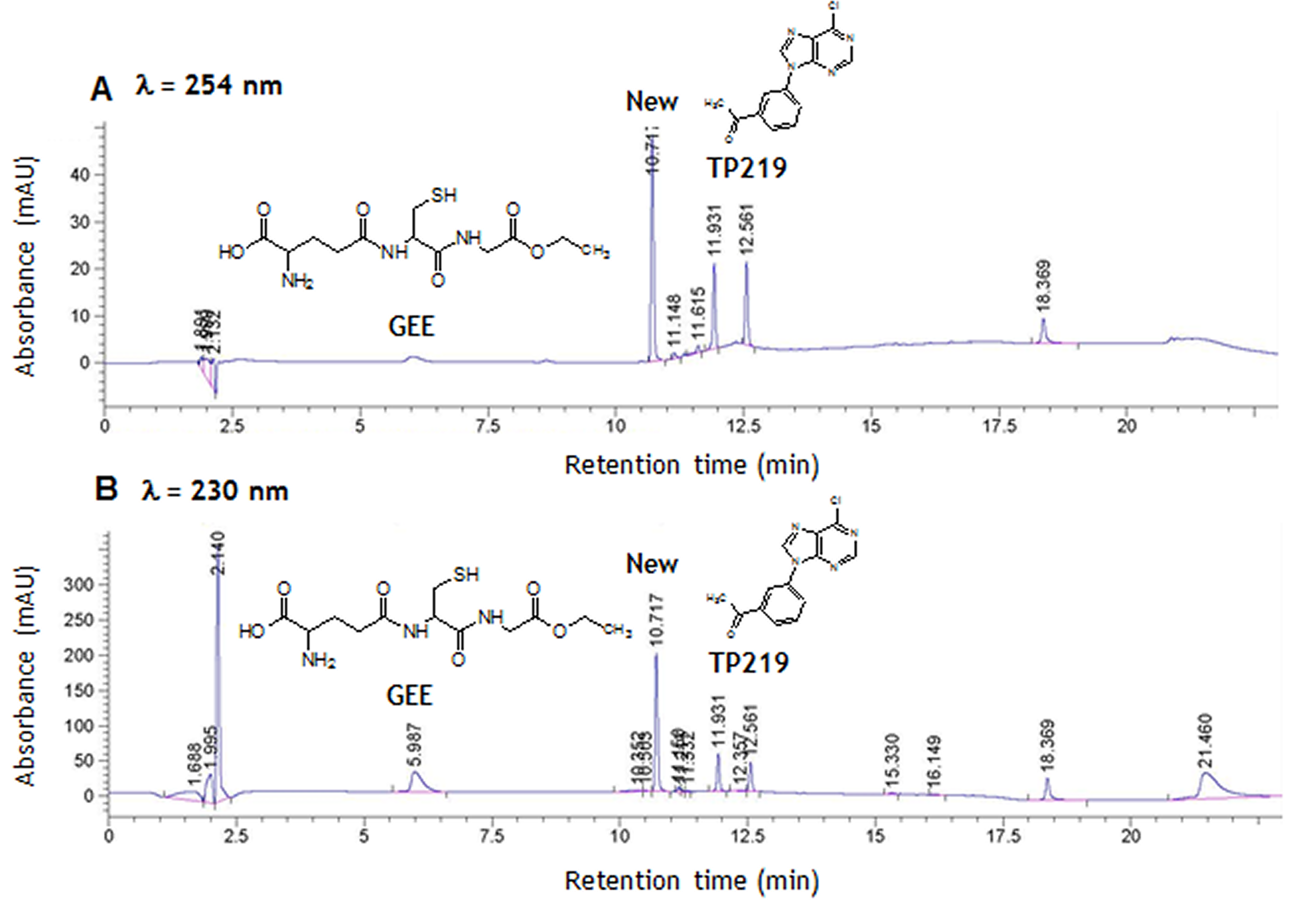

Supplement: Figure S2 — HPLC chromatogram of a new conjugate. TP219 and GEE were co-incubated for 6 h and analyzed by HPLC. Samples were analyzed at two wavelengths λ = 254 nm (A) and λ = 230 nm (B). (TIF) [file ppat.1004039.s002.tif]

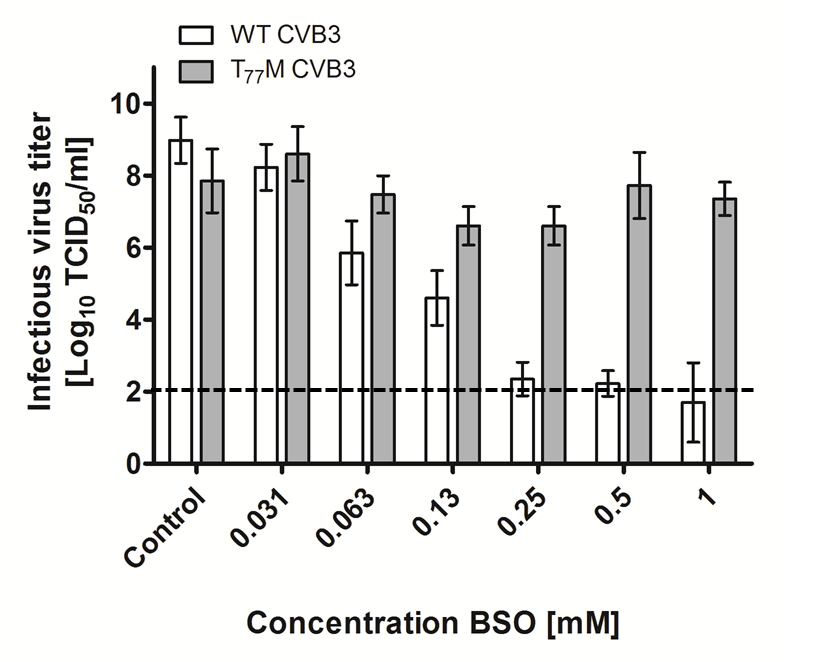

Supplement: Figure S3 — Cross-resistance to BSO. BSO inhibits formation of infectious virus particles of wild-type (white bars) but not of T77M (grey bars) CVB3 in a dose-dependent manner. Virus titers were calculated by endpoint titration and expressed as tissue culture 50% infectious dose per ml (Log10 TCID50/ml). Data are average values ± SD. (TIF) [file ppat.1004039.s003.tif]

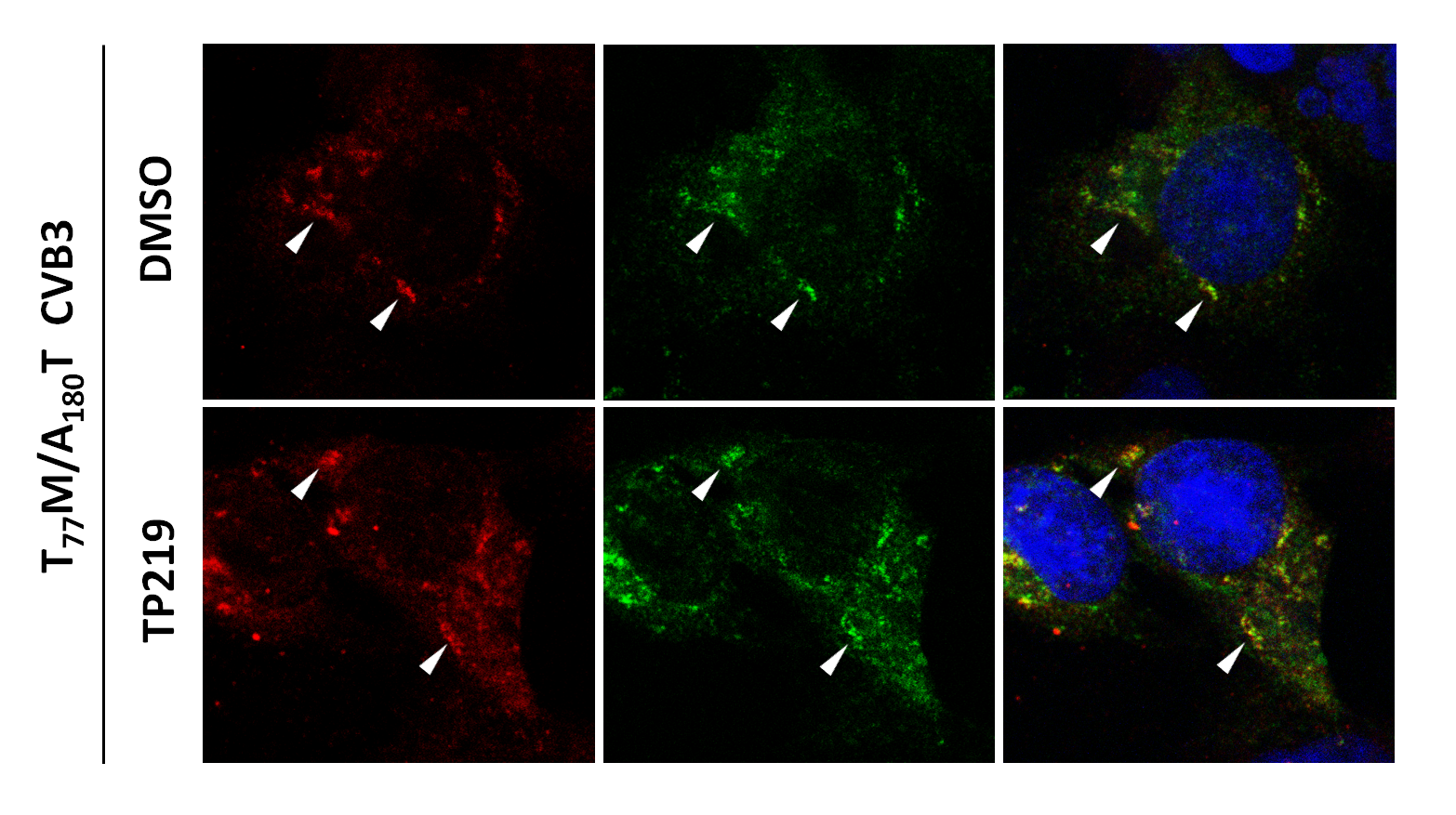

Supplement: Figure S4 — GSH depletion interferes with the interaction of VP1 and 2C. BGM cells were infected with T77M/A180T CVB3 at a MOI of 10, in the absence or the presence of 50 µM TP219. Cells were fixed with saponin 0.5% at 5 h p.i. and costained with antibodies targeting 2C (and Alexa Fluor 568-conjugated secondary antibody (red color)) and VP1 (and Alexa Fluor 488-conjugated secondary antibody (green color)). Regions of colocalization are indicated by arrows. (TIF) [file ppat.1004039.s004.tif]
